# Analysis and Fine Specificity of the HCMV-Specific Cell-Free and Cell-Associated Antibody-Dependent Cellular Phagocytosis (ADCP) Responses in Lung Transplant Recipients

**DOI:** 10.3390/ijms22158206

**Published:** 2021-07-30

**Authors:** Simone Eberhard, Hannes Vietzen, Irene Görzer, Peter Jaksch, Elisabeth Puchhammer-Stöckl

**Affiliations:** 1 Center for Virology, Medical University of Vienna, 1090 Vienna, Austria; simone.eberhard@meduniwien.ac.at (S.E.); irene.Goerzer@meduniwien.ac.at (I.G.); elisabeth.puchhammer@meduniwien.ac.at (E.P.-S.); 2 Division of Thoracic Surgery, Medical University of Vienna, 1090 Vienna, Austria; peter.jaksch@meduniwien.ac.at

**Keywords:** HCMV, ADCP, antibody, phagocytosis

## Abstract

Human Cytomegalovirus (HCMV) may cause severe infections in transplant recipients. HCMV-replication can be limited by HCMV-specific antibody responses. The impact of the antibody-dependent cellular phagocytosis (ADCP) on inhibition of HCMV-replication in natural infections has not been clarified. Therefore, we investigated the HCMV-specific ADCP response in a study cohort of lung-transplant recipients (LTRs) with different donor (D) and recipient (R) HCMV-serostatus. Follow-up plasma samples from 39 non/low-viremic and 36 highly viremic (>1000 HCMV copies/mL plasma) LTRs were collected for one (R+ LTRs) or two (D+/R− LTRs) years post-transplantation. The HCMV-specific ADCP responses were assessed by focal expansion assays (FEA) and flow-cytometry. In all LTRs, ADCP responses were detected against HCMV-infected cells and cell-free virions. When measured in fibroblasts as well as with cell-free virus, the HCMV-specific ADPC response was higher in LTRs than in HCMV-seropositive healthy controls. In D+/R− LTRs, a significant ADCP response developed over time after the receipt of an HCMV positive lung, and a level of <19 IE^+^ cells/focus in the FEA on fibroblasts was associated with further protection from high-level viremia. Taken together, a strong HCMV-specific ADCP response is elicited in transplant recipients, which may contribute to protection from high-level viremia in primary HCMV infection.

## 1. Introduction

Human Cytomegalovirus (HCMV) is a prevalent viral pathogen, which may not only cause severe and potentially life-threatening acute infections, but is also associated with long-term allograft injury and rejection in lung-transplant recipients (LTRs) (1). The risk to develop high-level HCMV-replication in LTRs depends on the allograft donor (D) and recipient (R) serostatus. While HCMV-seronegative recipients, who receive an organ from an HCMV-seropositive donor (D+/R−), have a high risk to acquire an HCMV-primary infection, reactivations and re-infections with different HCMV-strains may also occur in seropositive D−/R+ and D+/R+ recipients, respectively. HCMV-specific adaptive and innate immune responses may control virus replication, limit viral spread and prevent the development of HCMV-diseases [1].

HCMV-infections lead to a robust HCMV-specific neutralizing and non-neutralizing IgG1 and, to lower extent, also IgG3 antibody response, which mainly targets the viral glycoprotein B (gB) and the pentameric complex (PC) [2]. gB-specific neutralizing antibodies block infections of a broad range of cells, including fibroblasts, while, in contrast, PC-specific antibodies neutralize only the infection of epithelial, endothelial and myeloid cells. Neutralizing gB- and PC-specific antibodies are, however, unable to prevent the cell-to-cell spread of HCMV, which mainly occurs in epithelial and endothelial cells and results in effective HCMV-replication and transmission in these cell types [3,4]. In contrast, non-neutralizing antibodies may opsonize both, cell-free virions as well as HCMV-infected cells, and reduce the viral dissemination by different potent antibody-dependent effector functions, as antibody-dependent cellular cytotoxicity (ADCC) or phagocyte-mediated antibody-dependent cellular phagocytosis (ADCP) [2,5].

As so far, no licensed vaccine for HCMV exists for LTRs, there is generally a great interest in the identification of protective antibody-mediated HCMV-specific immune responses limiting natural HCMV-infections, which may guide the future vaccine design. Non-neutralizing antibodies recently gained interest as the gB/MF59 vaccine candidate elicited nearly exclusively a non-neutralizing antibody response in D+/R− solid organ-transplant (SOT) recipients [6,7]. It was shown that it was especially the non-neutralizing antibody response ADCP, which limited the viral replication in the patients and prevented to some extent the development of high-level HCMV viremia [5,6,7]. ADCP provides in general a potent mechanism for the clearance of cell-free virions as well as infected cells and stimulates downstream virus-specific immune responses by facilitating antigen presentation and by stimulating the secretion of pro-inflammatory cytokines [8]. ADCP is elicited by the recognition of antibody-opsonized HCMV-infected target cells or cell-free virions by Fcγ receptor (FcγR)-bearing phagocytes, which mainly include monocytes and macrophages and to a lesser extent also neutrophils, dendritic cells and eosinophils [9]. Monocytes mainly express FcγRI/CD64 and FcγRII/CD32, which show a high and medium affinity to IgG1 and IgG3 antibodies, respectively [10]. Upon FcγR stimulation, the antigen-antibody complex is engulfed by the phagocyte’s plasma membrane, followed by its subsequent degradation and clearance in the phagolysosome [11].

In the present study, we aimed to analyze the extent of the HCMV-specific ADCP during natural HCMV-infections to assess whether this particular non-neutralizing antibody mediated effector function is essential for protection against a high-level HCMV-viremia in LTRs.

## 2. Results

### 2.1. Study Cohort and Antibody Profile

In total, 75 LTRs (32 female, 43 male, median age: 53) were included in our study cohort. From all LTRs, 26 were seropositive and received a lung transplant from a seronegative donor (D−/R+), 31 were seropositive and received an allograft from a seropositive donor (D+/R+) and 18 were seronegative and received an organ from a seropositive donor (D+/R−). In total, 36 LTRs had a high-viremic HCMV-episode, exceeding > 1000 copies/mL blood, while 39 patients had a low- (N = 29, 20–487 copies/mL) or absent viral load (N = 10) in the blood. The patients’ and sample characteristics are presented in Supplementary Tables S1–S3.

To investigate the overall IgG subtype response to the immunodominant HCMV-encoded gB and PC, we have assessed the gB and PC specific IgG1 and 3 subclass antibody profile of the patients in the follow up samples. From all LTRs, 62 (82.6%) and 67 (89.3%) LTRs had detectable IgG1 or IgG3 antibodies against the viral gB, while 44 (58.6%) and 69 (92%) patients had detectable PC-specific IgG1 and IgG3 antibodies during the follow-up, respectively. The results are shown in detail in Supplementary Table S4.

### 2.2. Analysis of the HCMV-Specific ADCP Response against Infected Cells

To analyze whether the HCMV-specific ADCP response against HCMV-infected cells is associated with the protection from high-level HCMV-viremia, we first established a plasma-dependent focal expansion assay (FEA), based on the plaque growth of HCMV-TB40-BAC4-luc infected and HCMV-IE^+^ positive cells in the presence of plasma and the monocytic cell line THP-1. As shown in Supplementary Figure S1A–C addition of HCMV-seropositive plasma to the THP-1 cells limited the viral spread and reduced the plaque size in HCMV-seropositive, compared to HCMV-seronegative control persons in HCMV-infected fibroblasts (HFF) and epithelial (ARPE-19) cells.

**Figure 1:**
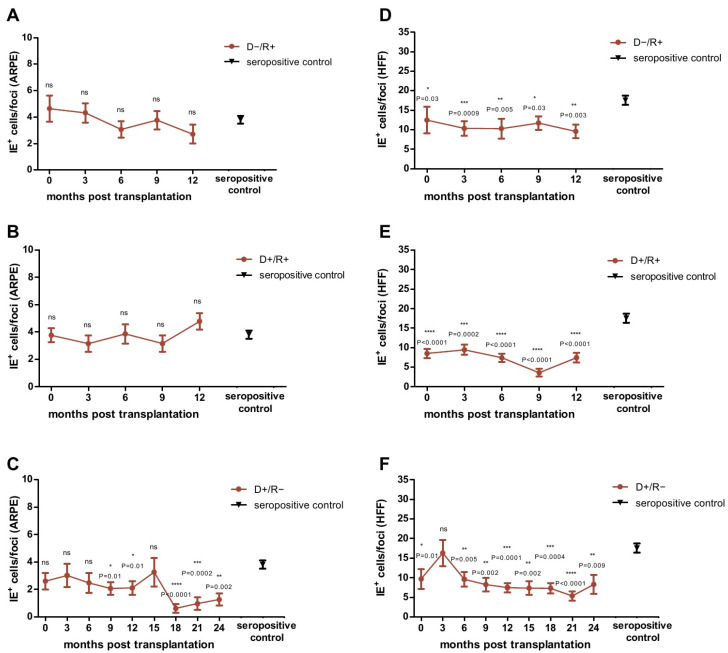
Analysis of the Focus Expansion Assay (FEA) on epithelial cells (ARPE) (**A**–**C**) and fibroblasts (HFFs) (**D**–**F**) with plasma obtained from D−/R+ (**A**,**D**), D+/R+ (**B**,**E**) and D+/R− (**C**,**F**) lung transplant recipients (LTRs) after lung transplantation (LTX). D−/R+ and D+/R+ LTRs were tested at 0, 3, 6, 9 and 12 months post LTX. D+/R− LTRs were tested at 0, 3, 6, 9, 12, 15, 18, 21 and 24 months post LTX. Data are shown as mean values (± SEM). The mean value of the HCMV-seropositive control cohort (N = 80) is indicated as black triangle (± SEM). Plaque sizes at each time point were compared between LTRs and seropositive control patients by ANOVA and Dunn’s post-test; *p* < 0.05 was considered significant. * *p* < 0.05; ** *p* < 0.01; *** *p* < 0.001; **** *p* < 0.0001. IE**^+^**: HCMV-immediate early positive cells.

We then tested each plasma sample obtained from the LTRs over time in the FEA on ARPE-19 cells and HFFs and compared the results obtained at each time point with that of the HCMV seropositive control cohort. As shown in Figure 1A,B, the ADCP response of D−/R+ and D+/R+ LTRs against infected ARPE-19 cells was comparable to that of the seropositive controls at all-time points. In contrast, we found a significantly better ADCP response in these patients against infected fibroblasts, for all-time points compared to controls (Figure 1D–E).

In D+/R− patients, undergoing primary infection with HCMV, the ADCP response against HCMV-infected cells developed over time as shown in the FEA. A slight increase in ADCP response against HCMV-infected ARPE-19 epithelial cells was observed from month 9 onward and became especially high from month 18 on. In contrast, a strong increase in ADCP against HCMV-infected fibroblasts was observed immediately after transplantation, and from month 6 after transplantation, to levels significantly higher than that of seropositive controls (Figure 1C,F).

### 2.3. HCMV-Specific ADCP Response in Viremic- and No/Low Viremic R+ LTRs

**Figure 2:**
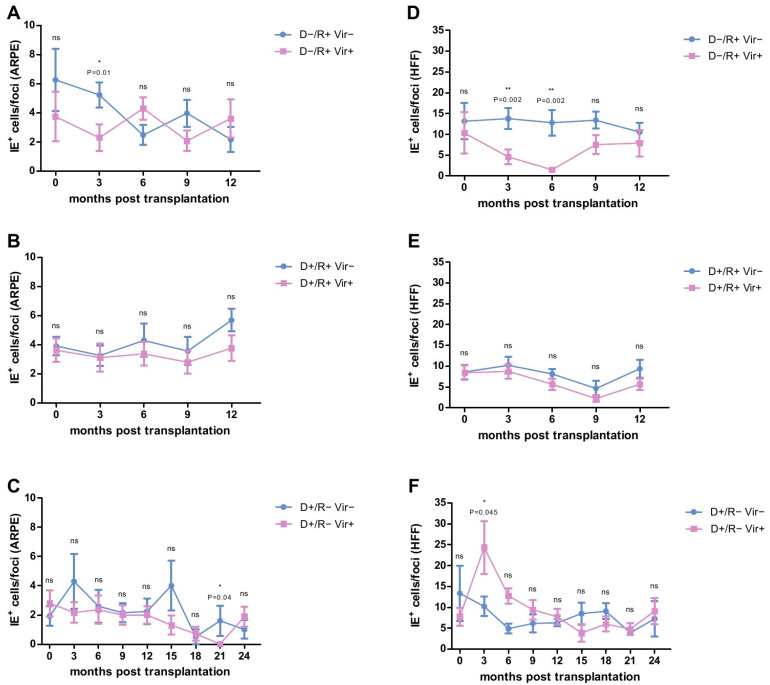
Analysis of the Focus Expansion Assay (FEA) on epithelial cells (ARPE) (**A**–**C**) and fibroblasts (HFFs) (**D**–**F**) with plasma obtained from D−/R+ (**A**,**D**), D+/R+ (**B**,**E**) and D+/R− (**C**,**F**) lung transplant recipients (LTRs) after lung transplantation (LTX). D−/R+ and D+/R+ LTRs were tested at 0, 3, 6, 9 and 12 months post LTX. D+/R− LTRs were tested at 0, 3, 6, 9, 12, 15, 18, 21 and 24 months post LTX. Data are shown as mean values (± SEM). Plaque sizes at each time point were compared between viremic (>1000 copies/mL) (pink) and non/low-viremic (<1000 copies/mL) (blue) LTRs by Mann-Whitney Test; *p* < 0.05 was considered significant. * *p* < 0.05; **, *p* < 0.01. IE**^+^**: HCMV-immediate early positive cells.

We then assessed whether the ADCP response against HCMV-infected cells differed between patients developing either at least one episode of high-level HCMV viremia (>1000 copies HCMV DNA /mL plasma) or showed no or only low levels of HCMV-replication over time. The data are presented in Figure 2A,D. Highly viremic D−/R+ patients had a significantly better ADCP response compared to non/low viremic D−/R+ LTRs. On HCMV-infected ARPE-19 cells this was observed at three months post-transplantation (Figure 2A), with a cut off level of <4 IE^+^ cells/per focus predicting subsequent high-level viremia (*p* = 0.038, OR = 10, PPV = 83.3%, NPV = 66.67%; χ2-Test; Supplementary Figure S2A). On HCMV-infected HFFs, a significantly better ADCP response was observed at month 3 to month 6 after transplantation (Figure 2D). A cut-off of <10 and <4 IE^+^ /per foci, respectively, was calculated (Supplementary Figure S2B,C), which predicted a subsequent high-level-viremia (3 months: *p* = 0.038, OR = 10, PPV = 83.3%, NPV = 66.67% and 6 months: *p* = 0.02, OR = 16.3, PPV = 100%, NPV = 71.4%; χ2-Test).

In contrast, in D+/R+ patients no significant differences were found between viremic and non/low-viremic patients in the FEA at any time point (Figure 2B,E).

### 2.4. HCMV-Specific ADCP Response in Viremic and No/Low Viremic D+/R− LTRs

We then analyzed whether there is a difference between the ADCP response of viremic and non/low viremic D+/R− LTRs, respectively. As shown in Figure 2C, we found a somehow better ADCP response against HCMV-infected ARPE-19 epithelial cells in viremic patients at 21 months post transplantation.

On fibroblasts however, D+/R− LTRs developing no/low viremia showed a significantly better ADCP response at 3 months post-transplantation, compared to viremic patients (Figure 2F). A cut-off of <19 IE^+^ cells/focus was calculated (Supplementary Figure S2D), which allowed to exclude a further occurrence of high-level HCMV replication in D+/R− LTRs (*p* = 0.06, OR = 10.11, PPV = 57.1%, NPV = 100%; χ2-Test).

### 2.5. Analysis of the HCMV-Specific ADCP Response against Cell-Free Virions

**Figure 3:**
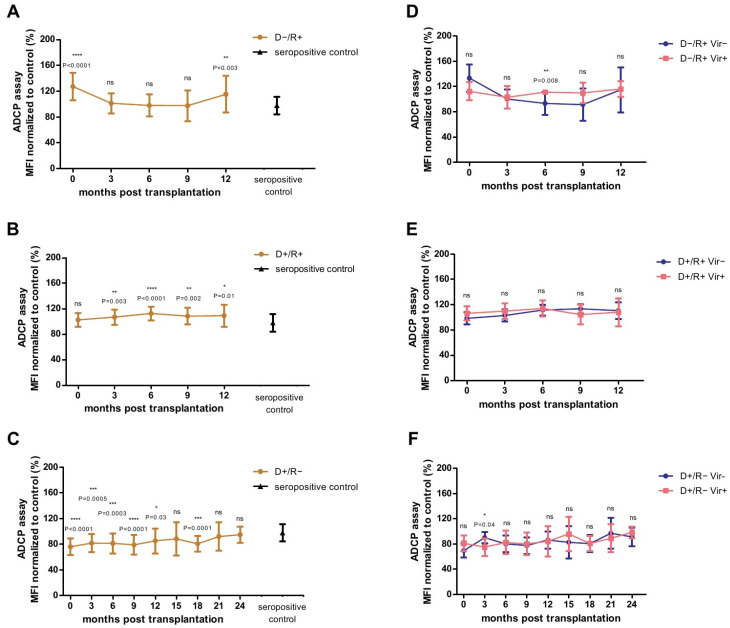
Analysis of the Antibody-Dependent Cellular Phagocytosis (ADCP)-Assay with plasma obtained from D−/R+ (**A**,**D**), D+/R+ (**B**,**E**) and D+/R− (**C**,**F**) lung transplant recipients (LTRs) after lung transplantation (LTX). D−/ R+ and D+/R+ LTRs were tested at 0, 3, 6, 9 and 12 months post-LTX. D+/R− LTRs were tested at 0, 3, 6, 9, 12, 15, 18, 21 and 24 months after LTX. Data are shown as mean values (±SD). Mean value of the HCMV-seropositive control cohort (N = 20) is indicated as black triangle (±SD) (**A**–**C**). The normalized mean fluorescence intensity (MFI) at each time point was compared with that of the seropositive control patients by ANOVA and Dunn’s post-test (**A**–**C**). MFI at each time point of viremic (>1000 copies/mL) (pink) LTRs was compared with that of non/low-viremic (<1000 copies/mL) (blue) LTRs by Mann-Whitney test (**D**−**F**); *p* < 0.05 was considered significant. * *p* < 0.05; ** *p* < 0.01; *** *p* < 0.001; **** *p* < 0.0001. MFI: mean fluorescence intensity.

Taken together, we found a significantly better ADCP response against HCMV-infected fibroblasts than in ARPE cells in all, D−/R+, D+/R+ and D+/R− LTRs. As infection of fibroblasts is associated with a more pronounced cell-free spread, compared to epithelial cells [4], we hypothesized that the HCMV-specific ADCP response against cell-free virus, in addition to HCMV-infected cells, plays an important role in limiting natural infections in LTRs. Therefore, we tested the plasma samples of the LTRs and the healthy seropositive controls in a whole HCMV virion phagocytosis assay, which is based on the detection of fluorescence labelled virus in THP-1 cells by flow-cytometry (Supplementary Figure S3). The results are shown in Figure 3. Overall, a higher ADCP response against cell-free virus was observed in D−/R+ LTRs than in seropositive control persons immediately after transplantation and after 12 months (Figure 3A), and in D+/R+ LTRs from 3 months post transplantation onwards (Figure 3B). In contrast, in D+/R− LTRs the ADCP response against cell-free virions was at most time points significantly lower than that of the seropositive controls (Figure 3C).

### 2.6. Association between HCMV-Specific Adcp Response against Cell-Free Virions and Viremia

We then compared the ADCP measured on cell-free HCMV-virions between high viremic and non/low viremic LTRs. As shown in Figure 3D, viremic D−/R+ showed a significant better ADCP response against cell-free HCMV virions at six months post-transplantation, compared to non/low-viremic LTRs. As shown in Supplementary Figure S4A a normalized MFI > 109% cut off was estimated which had a highly predictive potential to distinguish between future viremic and non/low viremic patients (*p* = 0.0006, OR = 35, PPV = 100%, NPV = 92.9%, χ2-Test).

In contrast, no significant differences were found between viremic and non/low viremic D+/R+ patients at any time point (Figure 3E).

As shown in Figure 3F, we found a significantly better ADCP response against cell-free virions in D+/R− patients developing no/low viremia in the follow up at three months post-transplantation, compared to highly viremic LTRs. A normalized cut-off of MFI >79% at three months post-transplantation predicted freedom from future viremia (Supplementary Figure S4B) (*p* = 0.0007, OR = 73.67, PPV = 88.9%, NPV = 100%, χ2-Test).

### 2.7. Correlation of the ADCP Response and the HCMV-Specific Antibody Titers

We further evaluated whether the better ADCP response against HCMV-infected cells and cell-free-virions in highly viremic, compared to non/low viremic patients observed at distinct time points is reflected by the level of HCMV-specific antibody titers. First, we correlated the gB- and PC-specific IgG1 and IgG3 titers of the D−/R+ patients with the plaque size, obtained from the FEA assay and the MFI, obtained from the cell-free flow cytometry assay, at the same time-point. Overall, no significant correlations could be detected.

We then also correlated the ADCP response of D+/R− LTRs against HCMV-infected fibroblasts and cell-free virions after three months post-transplantation to evaluate, whether the better ADCP response in non/low-viremic patients is reflected by higher HCMV-specific antibody titers. PC- and gB-specific IgG1 and IgG3 titers of D+/R− LTRs are shown in Supplementary Figure S5. A significant correlation was observed between higher gB IgG3 antibody titers and lower IE^+^ plaque sizes as well as higher MFI values in the whole virions phagocytosis assay at three months post-transplantation (Supplementary Figure S6, *p* = 0.01 and *p* = 0.04, respectively, Spearman’s rank correlation-Test).

### 2.8. Analysis of the ADCP Response over Time in Relation to Primary Infection

We finally also analyzed whether there is a relation between the episode of high-level viremia during primary infection in D+/R− and the level of the HCMV-specific ADCP response in the follow up. Therefore, we evaluated the HCMV-specific ADCP responses in D+/R− LTRs in the post-transplant follow-up and the data are presented in Supplementary Figure S7. We generally observed a significant increase in the HCMV-specific ADCP over time and in response to highly viremic episodes against HCMV-infected fibroblasts, ARPE-19 cells and cell-free virions (*p* = 0.04, *p* = 0.01, *p* = 0.003, respectively, Spearman’s rank correlation-Test).

## 3. Discussion

Our present study elucidates for the first time the HCMV-specific ADCP response in patients encountering HCMV primary infections or reactivations. Recent vaccine studies had brought attention to this specific antiviral response as the gB/MF59 vaccine candidate elicited nearly exclusively a non-neutralizing HCMV-specific ADCP response, which was associated with the protection from the development of high-level HCMV viremia in SOT recipients [5,6,7]. In contrast to the HCMV-specific neutralizing antibody response, the non-neutralizing HCMV-specific ADCP response does not only target cell-free HCMV-virions, but also HCMV-infected cells, and thus plays an important role in the HCMV-specific immune responses [12]. Now we analyzed the overall ADCP response against HCMV-infected cells and cell-free HCMV-virions in a study cohort of LTRs, in whom the HCMV-specific ADPC response could be set in relation to HCMV serostatus and viremia.

So far, limited studies focused on the HCMV-specific ADCP response. A recently published study demonstrated that healthy HCMV-infected individuals have a more robust ADCP response against cell-free HCMV virions, compared to gB/MF59 vaccinees [5]. We further revealed in the present study that there are substantial differences in the HCMV-specific ADCP responses between HCMV seropositive healthy individuals and transplant recipients. Seropositive LTRs, albeit undergoing immunosuppressive therapy developed a significantly better ADCP response, measured against infected fibroblasts, than not transplanted seropositive persons. Interestingly, we demonstrated in a recently published study, that also the non-neutralizing HCMV-specific NK cell mediated ADCC responses in LTRs exceeded the levels reached by non-transplanted seropositive persons [13]. Together with our current study, these data impressively demonstrate that HCMV-specific non-neutralizing ADCP and ADCC effector functions are early present and significantly shape the fine specificity of the HCMV-specific antibody-mediated immune responses.

As a main result, we further observed that an early increase in the HCMV-specific ADCP response against HCMV-infected fibroblasts and cell-free virions, can prevent to some degree the development of highly viremic episodes in D+/R− LTRs. Our data are of special interest, as these D+/R− patients undergoing primary infection by the HCMV positive graft are at high risk to develop severe HCMV- infection and HCMV-diseases [1]. We revealed that ADCP responses under a certain cut off level, at month 3 after transplantation were associated with development of high-level viremia in the follow up. Further prospective studies are needed to clarify the usefulness of an ADCP-mediated approach to identify patients at risk for high-level HCMV-viremia.

In the present study, only gB-specific IgG3 antibody titers correlated with the protective ADCP responses in D+/R− LTRs. These results are consistent with previous in vitro studies, which demonstrated that gB is not only abundant on cell-free HCMV virions but is also present on the surface of HCMV-infected fibroblasts and is thus a major target for non-neutralizing HCMV-specific antibodies [13,14,15]. It is thus likely, that gB-specific antibodies are the main effectors for the early HCMV-specific ADCP response. Our data suggest that an early gB-specific IgG3 mediated ADCP response against HCMV-infected fibroblasts and cell-free virions may prevent the dissemination of the virus within the allograft recipient and thus prevent the development of subsequent high-level virus replication. Overall, our data are in agreement with the gB/MF59 vaccine trial in SOT, which demonstrated that especially D+/R− patients with high gB-specific IgG titers were protected from high-level viremic episodes [6]. Combined, these and our data indicate, that ADCP mediating antibodies play an especially important role in limiting primary infections in transplant recipients.

In our D−/R+ study patients, we found, in contrast, a better HCMV-specific ADCP response in viremic, compared to non/low viremic LTRs, which demonstrates that the HCMV-specific ADCP response does not protect against recurrent HCMV-reactivations in these patients. Our data suggest that in these patients, pre-existing HCMV-specific ADCP responses may be rather boosted by recurrent and low level HCMV-reactivations, even prior to the emergence of high-viremic episodes. Notably, HCMV developed several immune evasion strategies to evade the antibody-mediated recognition of FcγR bearing effector cells. Viral decoy Fcγ-binding glycoproteins (vFcγRs) mediate an effective protection from HCMV-specific antibody opsonization and were recently demonstrated to prevent the HCMV-specific ADCC responses [16,17,18]. So far, no data are however available on the role of vFcγRs in the prevention of the HCMV-specific ADCP response against HCMV-infected cells and free-virions. Further studies are required to evaluate, whether especially reactivating HCMV manages to evade the HCMV-specific ADCP response, resulting to an accumulation of non-protective ADCP mediating antibodies.

In contrast to D−/R+ LTRs, we did not detect differences in the ADCP response between viremic and non/low-viremic D+/R+ LTRs, which demonstrates that the HCMV-specific ADCP response cannot prohibit reactivations and re-infections in these patients. Interestingly, recently published studies in gB/MF59 vaccinated R+ SOT recipients demonstrated that secondary exposure to novel HCMV-antigens leads to the preferred expansion of pre-existing, rather than antibodies specific against novel epitopes [19]. It is thus reasonable that re-infections in D+/R+ LTRs leads to the preferred expansion of pre-existing ADCP-mediating antibodies, which do not protect against re-infecting HCMV strains via ADCP. However, as a limitation of our study, we could not differentiate between reactivations and re-infections in D+/R+ LTRs and it remains an open question, whether the HCMV-specific ADCP response is different between R+ LTRs undergoing either high-level reactivation or re-infection.

Overall, we found in our study a distinct fine specificity of the HCMV-responses and highly variable patterns of the ADCP response against HCMV-infected cells and cell-free virions. In LTRs, we found a conserved stronger ADCP response against HCMV-infected fibroblasts than to HCMV-infected epithelial cells. These findings are in line with a recently published study, which demonstrated that the non-neutralizing HCMV-specific ADCC response against HCMV-infected fibroblasts is significantly stronger compared to that against infected epithelial cells [13]. Overall, the stronger non-neutralizing HCMV-specific ADCC and ADCP responses against fibroblasts may be due to varying stochastic expression of HCMV-encoded glycoproteins on the surface of different infected cell types [13]. HCMV is able to infect a broad cell range in vivo [20] and it is so far unknown, which infected cell types are preferably targeted by the HCMV-specific ADCP response. There are so far no studies available, which compare the expression of HCMV-encoded epitopes on the surface of different HCMV-infected cell types. Further studies are thus required to evaluate to the fine-specificity of HCMV-encoded targets for the HCMV-specific non-neutralizing antibody responses.

Remarkably, we observed that D+/R− LTRs had a substantial ADCP response against HCMV-infected fibroblasts, already immediately after transplantation, in the absence of a host derived HCMV-specific antibody response. As all D+/R− LTRs received an intravenous HCMV-hyperimmunglobulin preparation (Cytotect) immediately after transplantation, this may lead to the HCMV-specific ADCP responses detected in these patients early after TX. Even though the neutralizing and ADCC-inducing capacity of Cytotect is well established [13,21], no data are so far available on the effects of HCMV-hyperimmunglobulins on the HCMV-specific ADCP response. Nonetheless, Cytotect contains large amounts of gB-specific antibodies, which may lead to the high ADCP response [15,21]. However, further studies are needed to analyse in detail the effect of Cytotect and other HCMV-hyperimmunglobulin preparations on the ADCP response early after transplantation.

We observed differences in ADCP response over time between highly viremic and non-viremic patients, which were statistically significant only at single time points in the follow up. This variability in the HCMV-specific ADCP response is in agreement with previous data which demonstrated that the level and fine specificity of other HCMV-specific non-neutralizing antibody responses and effector functions substantially vary over time after lung transplantation [13]. Further and larger studies are needed, to confirm the present findings and to reveal the confounding factors influencing the HCMV-specific non-neutralizing antibody responses.

Overall, our data are of special interest, as all LTRs received a strong immunosuppressive induction and maintenance therapy consisting of the anti-CD52 monoclonal antibody alemtuzumab, tacrolimus and corticosteroid, which leads on one hand to the depletion and on the other hand to a deferred immune reconstruction of HCMV-specific T-cells [22]. While thus HCMV-specific T-cell responses are severely impaired in LTRs, ex vivo studies showed that there is only a minimal effect of alemtuzumab, tacrolimus and corticosteroid treatments on counts and antiviral functions of monocytes [23,24,25]. It is thus likely that HCMV-specific ADCP plays a particular important role in the HCMV-specific antibody response, especially in transplant recipients.

As a major limitation of our study, we performed our cell-based and cell-free ADCP assays only with the laboratory TB40-BAC4-luc strain, which has conserved features of clinical wild−type HCMV-strains, such as infection of epithelial cells and cell-to-cell spread [20]. Wild−type HCMV-strains, however, encode for a remarkable diversity of the gB and PC, which may influence the elicited HCMV-specific antibody responses [26]. Thus, further studies are needed to analyze whether such variations may have a significant impact on the patients’ ADCP response.

In summary, we reveal in the present study the role of ADCP responses during natural HCMV-infections and provide evidence that a strong HCMV-specific ADCP response is elicited in patients after transplantation. Especially in patients at risk for primary HCMV infection, HCMV-specific ADCP responses may significantly contribute to protection from high-level HCMV replication and thereby form severe disease.

## 4. Material and Methods

### 4.1. Patients and Samples

In this study 75 patients who received a lung transplant between 2013 and 2018 at the Medical University of Vienna were included. Plasma samples of all patients were collected in three-month intervals starting immediately after transplantation and ending either 12 (for D−/R+ and D+/R+ patients) or 24 months (for D+/R− patients) post lung transplantation (LTX). All patients received immunosuppressive therapy with alemtuzumab, tacrolimus and corticosteroids. (Val-)Ganciclovir prophylaxis was given for 3 months in D−/R+ and D+/R+ patients or for 12 in D+/R− patients. HCMV-hyperimmunoglobulin (Cytotect, 100 units/kg) was administered once weekly, for 4 weeks post transplantation. Patients were screened by quantitative HCMV-PCR (qPCR) weekly for two months, and afterwards monthly to bimonthly. At a level of >1000 copies HCMV DNA/mL in blood, pre-emptive (Val-)Ganciclovir treatment was initiated for two weeks in all patients.

As controls, plasma samples from 80 HCMV-seropositive and 20 HCMV-seronegative individuals, which had no history of transplantation, were included.

From all plasma samples, HCMV gB- and PC-specific IgG1 and IgG3 antibodies were determined by ELISA as described in detail before [13]. Samples from HCMV-seronegative controls were used to determine the 95% CI cut-off for gB and PC IgG1 and IgG3 antibody titers as also described before [27].

### 4.2. Cells and Virus

Human foreskin fibroblasts (HFF) and arising retinal pigment epithelial cells (ARPE-19) were cultured in minimum essential medium (MEM) + 10% FBS (Sigma-Aldrich, St. Louis, MO, USA). THP-1 cells were maintained in RPMI-1640, including 10% FBS, 30 nM mercaptoethanol and 1% Pen-Strep (Sigma-Aldrich, St. Louis, MO, USA). The HCMV-laboratory strain TB40-BAC4-luc [4] (kindly provided by Barbara Adler, Max von Pettenkofer−Institute of Virology, Munich, Germany) was used for all experiments. Virus stocks were prepared in HFFs and enriched by ultracentrifugation (30,000 U/min, 90 min, 10 °C). The multiplicity of infection (MOI) was determined by immunofluorescence microscopy as described in detail before [13].

### 4.3. Focus Expansion Assay (FEA)

HFFs were infected with a MOI of 0.5, incubated 3 days at 37 °C and subsequently stored at −80 °C in MEM + 10% DMSO. FEAs were performed with HFF and ARPE-19 cells (1.5 × 10^4^ cells well), and a 400-fold excess of non-infected to infected HFF seeded on 96-well plates and incubated at 37 °C overnight. Patient or control plasma samples were diluted 1:10 and added together with 6 × 10^4^ THP-1 cells to each well of HFF or ARPE-19 cells. After co-culturing for 3 days at 37 °C, cells were washed with 1 × PBS + 0.05% Tween20 and fixed with 80% acetone. Infected cells were stained by immunofluorescence staining of the viral immediate early (IE) antigen using monoclonal mouse anti IE^+^ antibody (1:1000, Bio-Rad, Hercules, CA, USA), as described before [13]. IE^+^ cells were counted by immunofluorescence microscopy using the LAS X software (Leica Microsystems, Wetzlar, Germany). A plaque was defined as an assembly of three or more IE^+^ cells.

### 4.4. Whole HCMV Virion Phagocytosis Assay

The whole virion phagocytosis was measured using a recently published protocol [5]. In brief, 120 µL of a 155 infectious particles/µL TB40-BAC4-luc stock was fluorescently labelled with AF647 NHS-Ester 234, according to the manufacture’s instruction (Invitrogen, Waltham, MA, USA). After incubating at room temperature for 1 h with constant agitation, reaction was quenched with 1 M Tris-HCl (pH 8.0). Patient’s plasma was diluted 1:10 in 1 × PBS + 0.1% Tween20 and 10 µL was combined with 50 µL fluorophore-conjugated virus as well as 10 µL RPMI medium. After 2 h incubation at 37 °C, 35,000 THP-1 cells were added and centrifuged at 1200× *g*, 4 °C for 1 h. Cells were then incubated for 1 h at 37 °C, washed twice with 1 × PBS and fixed with fixation solution (Fix&Perm kit, Life Technologies, Carlsbad, CA, USA) according to the manufacture’s instruction. Cells were stained with mouse anti-human BV421-CD14 mAB (BD Biosciences, Franklin Lakes, NJ, USA). Cells were analysed on BD FACSCanto II flow cytometer using the BD FACSDiva Software (BD, Franklin Lakes, NJ, USA). Dead cells were excluded with 7-aminoactinomycin D (7-AAD, BD Biosciences, Franklin Lakes, NJ, USA) and mean fluorescence intensity (MFI) was calculated for each sample and normalized to one control patient. A 95% CI cut-off was defined using 20 HCMV-seronegative control sera, as described before [27].

### 4.5. Statistical Analysis

ANOVA and Dunn’s post-test were used to compare HCMV-seropositive control patients and LTRs at different time points. An unpaired, two-tailed, non-parametric t test (Mann-Whitney test) was used to compare highly viremic (>1000 copies/mL) and non/low-viremic patients (<1000 copies/mL). χ2-Test was used to compare frequency distributions between highly viremic and non/low-viremic LTRs. Odds ratio (OR), positive predictive values (PPV) and negative predictive values (NPV) were used to calculate the risk for a future HCMV-viremia. Linear regression and the non-parametric two-tailed Spearman’s rank correlation test were used for evaluation of the correlation between antibody-titers and ADCP responses as well as the correlation of the HCMV-specific ADCP responses in the post-transplant follow-up. A *p*-value of <0.05 was considered significant. All statistical analyses were performed using GraphPad Prism 5 (Graphpad Software, Inc., San Diego, CA, USA. The study was approved by the local ethics committee (EK No. 1120/2020).

## Data Availability

The data presented in this study are available on request from the corresponding author. The data are not publicly available due to privacy restrictions.

## Supplementary Materials

The following are available online at https://www.mdpi.com/article/10.3390/ijms22158206/s1.

## Abbreviations

ADCC	antibody-dependent cellular cytotoxicity
ADCP	antibody-dependent cellular phagocytosis
D−	donor HCMV-seronegative
D+	donor HCMV-seropositive
FcγR	Fcγ receptors
FEA	focal expansion assay
gB	glycoprotein B
HCMV	human cytomegalovirus
HFF	human foreskin fibroblasts
IE antigen	immediate early antigen
LTX	lung transplantation
LTR	lung transplant recipients
MFI	mean fluorescent intensity
MOI	multiplicity of infection
NK cell	natural killer cell
NPV	positive predictive values
OR	odds ratio
PC	pentameric complex
PPV	negative predictive values
R−	recipient HCMV-seronegative
R+	recipient HCMV-seropositive
SOT	solid organ transplantation
TX	transplantation

## References

[1] Razonable R.R., Humar A. (2019). Cytomegalovirus in solid organ transplant recipients—guidelines of the American Society of Transplantation Infectious Diseases Community of Practice. Clin. Transplant..

[2] Gomes A.C., Griffiths P.D., Reeves M.B. (2019). The humoral immune response against the gB vaccine: Lessons learnt from protection in solid organ transplantation. Vaccines.

[3] Jacob C.L., Lamorte L., Sepulveda E., Lorenz I.C., Gauthier A., Franti M. (2013). Neutralizing antibodies are unable to inhibit direct viral cell-to-cell spread of human cytomegalovirus. Virology.

[4] Scrivano L., Sinzger C., Nitschko H., Koszinowski U.H., Adler B. (2011). HCMV spread and cell tropism are determined by distinct virus populations. PLoS Pathog..

[5] Nelson C.S., Huffman T., Jenks J.A., de la Rosa E.C., Xie G., Vandergrift N., Permar S.R. (2018). HCMV glycoprotein B subunit vaccine efficacy mediated by nonneutralizing antibody effector functions. Proc. Natl. Acad. Sci. USA.

[6] Griffiths P.D., Stanton A., McCarrell E., Smith C., Osman M., Harber M., Burroughs A.K. (2011). Cytomegalovirus glycoprotein-B vaccine with MF59 adjuvant in transplant recipients: A phase 2 randomised placebo-controlled trial. Lancet.

[7] Baraniak I., Kropff B., Ambrose L., McIntosh M., McLean G.R., Pichon S., Reeves M.B. (2018). Protection from cytomegalovirus viremia following glycoprotein B vaccination is not dependent on neutralizing antibodies. Proc. Natl. Acad. Sci. USA.

[8] Tay M.Z., Wiehe K., Pollara J. (2019). Antibody-dependent cellular phagocytosis in antiviral immune responses. Front. Immunol..

[9] Rosales C., Uribe-Querol E. (2017). Phagocytosis: A fundamental process in immunity. BioMed Res. Int..

[10] Nimmerjahn F., Ravetch J.V. (2008). Fcγ receptors as regulators of immune responses. Nat. Rev. Immunol..

[11] Uribe-Querol E., Rosales C. (2020). Phagocytosis: Our current understanding of a universal biological process. Front. Immunol..

[12] Nelson C.S., Herold B.C., Permar S.R. (2018). A new era in cytomegalovirus vaccinology: Considerations for rational design of next-generation vaccines to prevent congenital cytomegalovirus infection. Npj Vaccines.

[13] Vietzen H., Görzer I., Honsig C., Jaksch P., Puchhammer-Stöckl E. (2020). Human Cytomegalovirus (HCMV)-Specific Antibody Response and Development of Antibody-Dependent Cellular Cytotoxicity Against HCMV After Lung Transplantation. J. Infect. Dis..

[14] Brait N., Stögerer T., Kalser J., Adler B., Kunz I., Benesch M., Görzer I. (2020). Influence of human cytomegalovirus glycoprotein O polymorphism on the inhibitory effect of soluble forms of trimer-and pentamer-specific entry receptors. J. Virol..

[15] Britt W.J., Vugler L.G. (1989). Processing of the gp55-116 envelope glycoprotein complex (gB) of human cytomegalovirus. J. Virol..

[16] Corrales-Aguilar E., Hoffmann K., Hengel H. (2014). CMV-encoded Fcγ receptors: Modulators at the interface of innate and adaptive immunity. Seminars in Immunopathology.

[17] Corrales-Aguilar E., Trilling M., Hunold K., Fiedler M., Le VT K., Reinhard H., Hengel H. (2014). Human cytomegalovirus Fcγ binding proteins gp34 and gp68 antagonize Fcγ receptors I, II and III. PLoS Pathog..

[18] Kolb P., Hoffmann K., Sievert A., Reinhard H., Merce-Maldonado E., Le-Trilling VT K., Hengel H. (2021). Human cytomegalovirus antagonizes activation of Fcγ receptors by distinct and synergizing modes of IgG manipulation. Elife.

[19] Baraniak I., Kern F., Holenya P., Griffiths P., Reeves M. (2019). Original antigenic sin shapes the immunological repertoire evoked by human cytomegalovirus glycoprotein B/MF59 vaccine in seropositive recipients. J. Infect. Dis..

[20] Sinzger C., Digel M., Jahn G. (2008). Cytomegalovirus cell tropism. Hum. Cytomegal..

[21] Schampera M.S., Schweinzer K., Abele H., Kagan K.O., Klein R., Rettig I., Hamprecht K. (2017). Comparison of cytomegalovirus (CMV)-specific neutralization capacity of hyperimmunoglobulin (HIG) versus standard intravenous immunoglobulin (IVIG) preparations: Impact of CMV IgG normalization. J. Clin. Virol..

[22] Jaksch P., Ankersmit J., Scheed A., Kocher A., Muraközy G., Klepetko W., Lang G. (2014). Alemtuzumab in lung transplantation: An open-label, randomized, prospective single center study. Am. J. Transplant..

[23] Freedman M.S., Kaplan J.M., Markovic-Plese S. (2013). Insights into the mechanisms of the therapeutic efficacy of alemtuzumab in multiple sclerosis. J. Clin. Cell. Immunol..

[24] Kannegieter N.M., Hesselink D.A., Dieterich M., Kraaijeveld R., Rowshani A.T., Leenen P.J., Baan C.C. (2017). The effect of tacrolimus and mycophenolic acid on CD14+ monocyte activation and function. PLoS ONE.

[25] Ehrchen J.M., Roth J., Barczyk-Kahlert K. (2019). More than suppression: Glucocorticoid action on monocytes and macrophages. Front. Immunol..

[26] Foglierini M., Marcandalli J., Perez L. (2019). HCMV envelope glycoprotein diversity demystified. Front. Microbiol..

[27] Frey A., Di Canzio J., Zurakowski D. (1998). A statistically defined endpoint titer determination method for immunoassays. J. Immunol. Methods.

