# Supplementary material for: Analysis and Fine Specificity of the HCMV-Specific Cell-Free and Cell-Associated Antibody-Dependent Cellular Phagocytosis (ADCP) Responses in Lung Transplant Recipients
Source: Int J Mol Sci. 2021 Jul 30;22(15):8206. doi: 10.3390/ijms22158206 (PMC8348747; doi:10.3390/ijms22158206)
Supplement: Supplementary file 1 [file ijms-22-08206-s001.zip › ijms-1291664-supplementary.pdf]

Supplementary Material

Analysis and Fine Specificity of the HCMV-Specific Cell-Free and Cell-Associated Antibody-Dependent Cellular Phagocytosis (ADCP) Responses in Lung Transplant Recipients

Supplementary Tables

Table S1. Characteristics of D−/R+ patients.

	D−/R+ Lung-Transplant Recipients (N = 26)	
	**Viremic**<br>**N = 9 (34.6%)**	**Non/Low-Viremic**<br>**N = 17 (65.4%)**
Median Age at LTX (range)	50 (37–69)	55 (26–66)
Gender	Female: N = 3 (33.3%)<br>Male: N = 6 (66.7%)	Female: N = 9 (53%)<br>Male: N = 8 (47%)
Time points: median days post LTX (range)		
T0: Median days (min–max)	1 (0–2)	1 (0–3)
T1: Median days (min–max)	92.5 (82–105)	90 (77–106)
T2: Median days (min–max)	180 (176–188)	186.5 (159–200)
T3: Median days (min–max)	280 (267–293)	283.5 (251–297)
T4: Median days (min–max)	363 (352–366)	369 (361–391)
1st viremic episode [1]	N = 9 (100%)	
Median days post LTX (range)	135 (41–364)	
Median viral load (copies/mL) (range)	2.53×10^3 (1.14×10^3–1.04×10^4)	

D+, HCMV-seropositive donor; D−, HCMV-seronegative donor; HCMV, human cytomegalovirus; LTX, lung transplantation; R+, HCMV-seropositive recipient; R−, HCMV-seronegative recipient. [1] Four patients had a 2nd viremic episode (median 1.69×10^3 copies/mL (1.05×10^3–1.29×10^4 copies/mL)) after median 205.5 days (135–258 days) within the follow-up.

Table S2. Characteristics of D+/R+ patients.

	D+/R+ Lung-Transplant Recipients (N = 31)	
	**Viremic N = 16 (51.6%)**	**Non/Low-Viremic N = 15 (48.4%)**
Median Age at LTX (range)	58.5 (18–65)	60 (21–68)
Gender	Female: N = 8 (50%)<br>Male: N = 8 (50%)	Female: N = 5 (33.3%)<br>Male: N = 10 (66.7%)
Time points: median days post LTX (range)		
T0: Median days (min–max)	0 (0–3)	1 (0–4)
T1: Median days (min–max)	93 (76–115)	93 (84–103)
T2: Median days (min–max)	182 (155–200)	180 (161–194)
T3: Median days (min–max)	288 (251–322)	276 (247–291)
T4: Median days (min–max)	390.5 (343–403)	365 (354–389)
1st viremic episode [1]	N = 16 (100%)	
Median days post LTX (range)	133.5 (16–254)	
Median viral load (copies/mL) (range)	2.64×10^3 (1.07×10^3–1.12×10^5)	

D+, HCMV-seropositive donor; D−, HCMV-seronegative donor; HCMV, human cytomegalovirus; LTX, lung transplantation; R+, HCMV-seropositive recipient; R−, HCMV-seronegative recipient. [1] Three patients had a 2nd viremic episode (median 3.3×10^3 copies/mL (2.9×10^3–1.48×10^4 copies/mL)) after median 207 days (134–262 days) within the follow-up.

Table S3. Characteristics of D+/R− patients.

	**D+/R− Lung-Transplant Recipients (N = 18)**	
	**Viremic N = 11 (61.1%)**	**Non/Low-Viremic N = 7 (38.9%)**
Median Age at LTX (range)	31 (21-63)	45 (22-61)
Gender	Female: N = 4 (36.4%)<br>Male: N = 7 (63.6%)	Female: N = 3 (42.9%)<br>Male: N = 4 (57.1%)
Time points: median days post LTX (range)		
T0: Median days (min–max)	1 (0–2)	2 (1–6)
T1: Median days (min–max)	89.5 (78–99)	87.5 (80–117)
T2: Median days (min–max)	182 (157–215)	196 (177–209)
T3: Median days (min–max)	275 (252–296)	265 (254–293)
T4: Median days (min–max)	367 (343–398)	370 (359–380)
T5: Median days (min–max)	468 (457–480)	466 (446–477)
T6: Median days (min–max)	534 (521–593)	544 (511–583)
T7: Median days (min–max)	654 (592–684)	648 (595–678)
T8: Median days (min–max)	709 (675–717)	714 (691–776)
1st viremic episode [1]	N = 11 (100%)	
Median days post LTX (range)	179 (65–469)	
Median viral load (copies/mL) (range)	3.89×10^3 (1.16×10^3–8.16×10^4)	

D+, HCMV-seropositive donor; D−, HCMV-seronegative donor; HCMV, human cytomegalovirus; LTX, lung transplantation; R+, HCMV-seropositive recipient; R−, HCMV-seronegative recipient. [1] Two patients had a 2nd viremic episode (median 7.49×10^3 copies/mL (1.6×10^3–1.3×10^4 copies/mL)) after median 433.5 days (420–447 days) within the follow-up.

Table S4. Serology of Lung-Transplant Recipients.

Serostatus	Study Cohort					
	**D−/R+ Lung-Transplant Recipients (N = 26)**		**D+/R+ Lung-Transplant Recipients (N = 31)**		**D+/R− Lung-Transplant Recipients (N = 18)**	
	**Viremic N = 9 (34.8%)**	**Non/Low-Viremic N = 17 (65.4%)**	**Viremic N = 16 (51.6%)**	**Non/Low-Viremic N = 15 (48.4%)**	**Viremic N = 11 (61.1%)**	**Non/Low-Viremic N = 7 (38.9%)**
gB-specific IgG1	N = 8 (88.94%)	N = 15 (88.24%)	N = 13 (81.25%)	N = 13 (86.68%)	N = 9 (81.82%)	N = 4 (57.14%)
gB-specific IgG3	N = 7 (77.78%)	N = 16 (94.12%)	N = 15 (93.75%)	N = 1 (93.33%)	N = 9 (81.82%)	N = 6 (85.71%)
PC-specific IgG1	N = 3 (33.33%)	N = 15 (88.24%)	N = 9 (56.25%)	N = 12 (80%)	N = 11 (100%)	N = 4 (57.14%)
PC-specific IgG3	N = 8 (88.94%)	N = 14 (82.35%)	N = 16 (100%)	N = 15 (100%)	N = 11 (100%)	N = 5 (71.42%)

D+, HCMV-seropositive donor; D−, HCMV-seronegative donor; gB: glycoprotein B; HCMV, human cytomegalovirus; LTX, lung transplantation; PC: pentameric complex; R+, HCMV-seropositive recipient; R−, HCMV-seronegative recipient.

Supplementary Figures

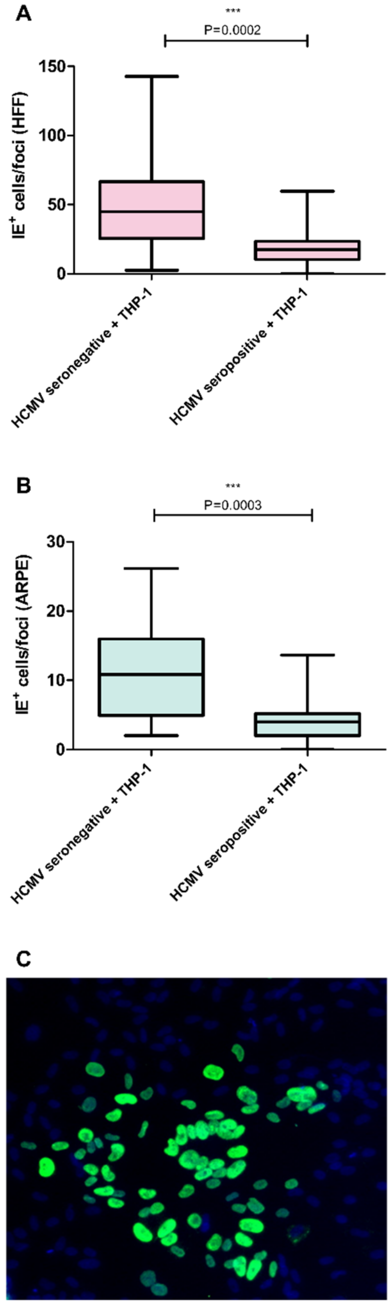

Figure S1. Establishment of the plasma-dependent focus expansion assay (FEA) with THP-1 cells on (**A**) Human Foreskin Fibroblasts (HFF) (**B**) or epithelial cells (ARPE) with HCMV-seropositive (N = 80) and HCMV-seronegative (N = 20) plasma samples. Data are shown as mean values (min–max). Plaque sizes between different experimental settings were compared by Mann Whitney-Test. (**C**) Representative example of a HCMV focus on human foreskin fibroblasts (HFF) in the presence of THP-1 cells and a HCMV-seronegative control plasma. $p < 0.05$ was considered significant. ***, $p < 0.001$. IE^{+}, HCMV-immediately early positive cells.

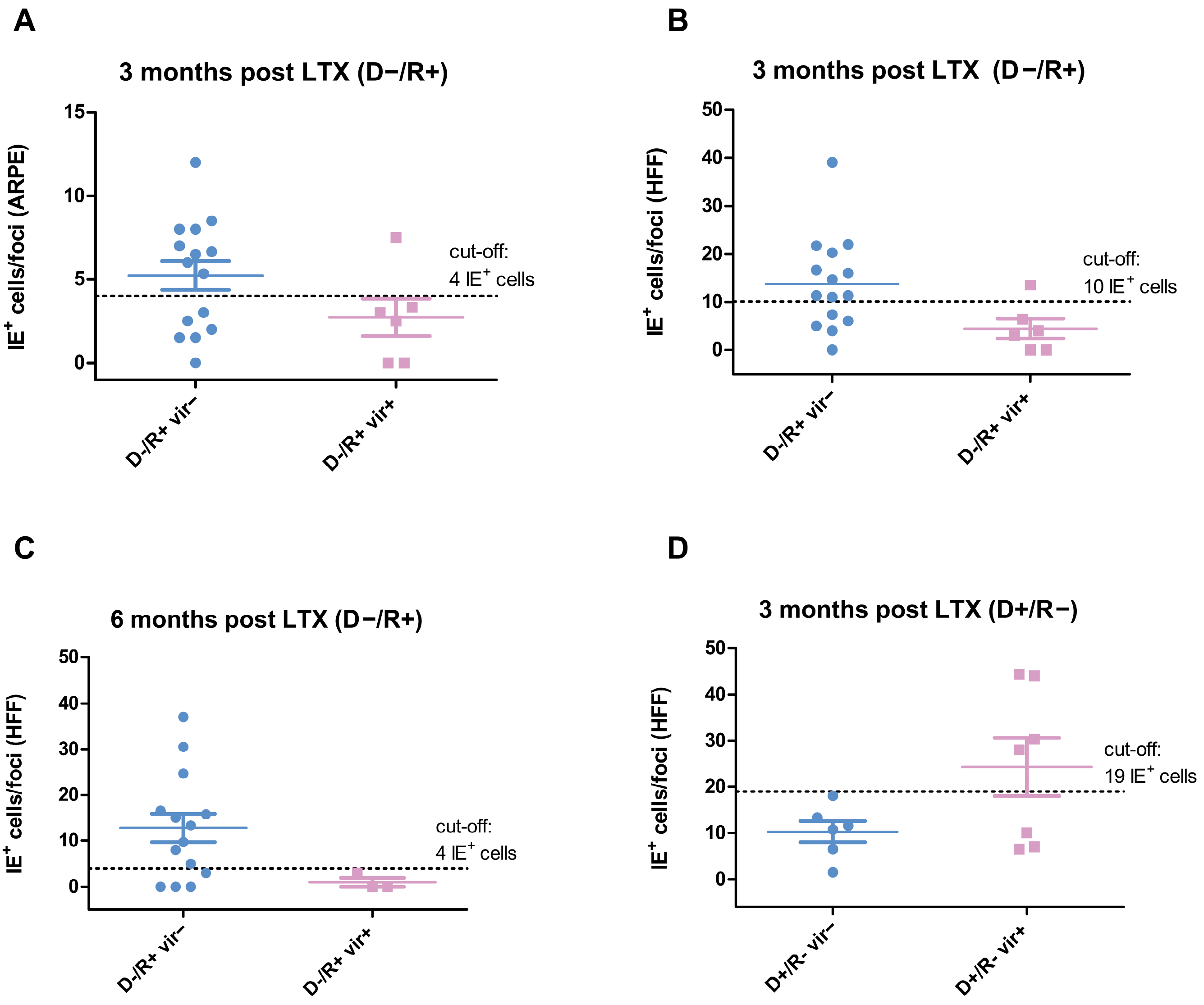

Figure S2. Estimated cut-offs for calculation of positive predictive values (PPV) and negative predictive values (NPV) in either epithelial cells (ARPE) (**A**) or fibroblasts (HFF) (**B**–**D**) in viremic (pink) and non viremic (blue) D−/R+ (**A**,**C**) and D+/R− (**D**) lung transplant recipients (LTRs) at several time points post lung transplantation (LTX). The dashed black line indicates the set cut-off.

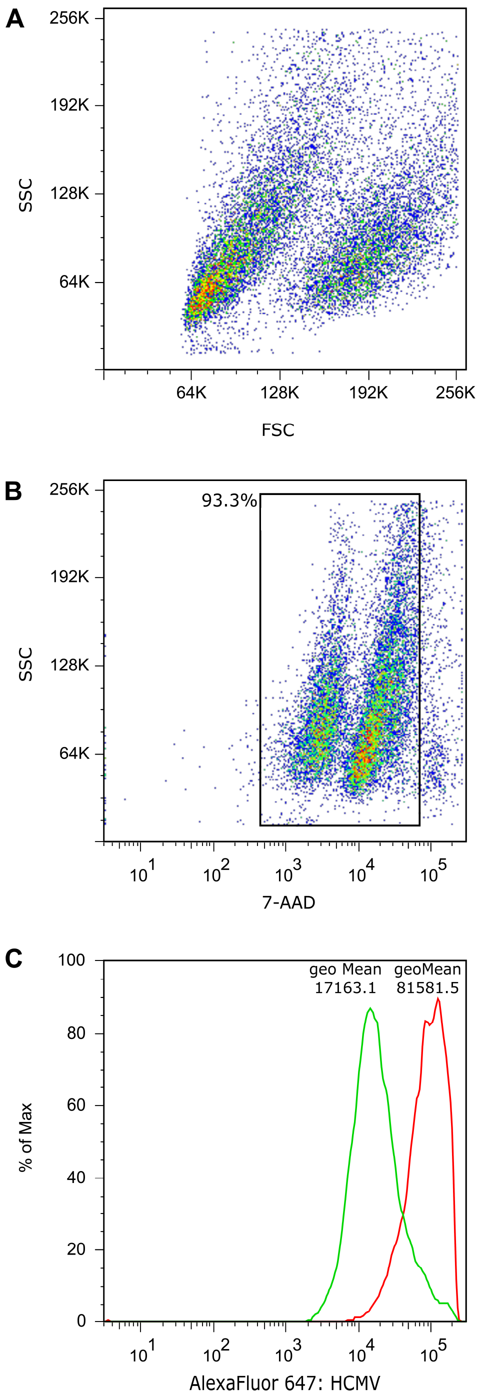

Figure S3. Representative example of the gating strategy and determination of life (7-AAD[low]) THP-1 cells (**A**,**B**) stimulated with TB40E cell-free virions and either a HCMV-seropositive (red) or a HCMV-seronegative (green) control plasma (**C**).

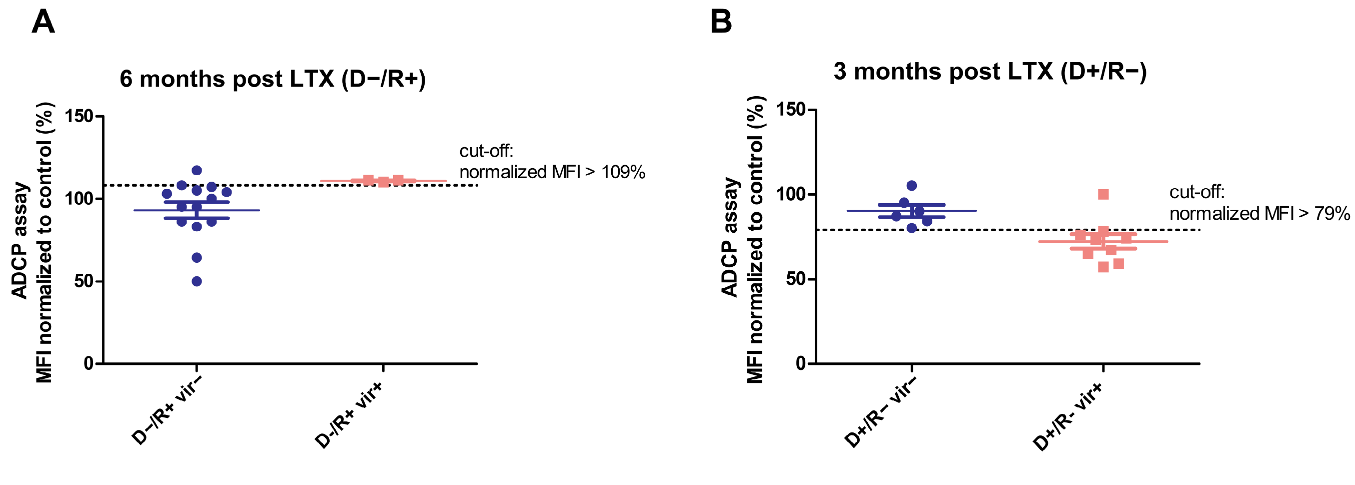

Figure S4. Estimated cut-offs for calculation of positive predictive values (PPV) and negative predictive values (NPV) in viremic (pink) and non/viremic (blue) D−/R+ lung transplant recipients (LTRs) at 6 months post lung transplantation (LTX) (**A**) and D+/R− LTRs at 3 months post lung transplantation (LTX) (**B**). The dashed black line indicates the set cut-off.

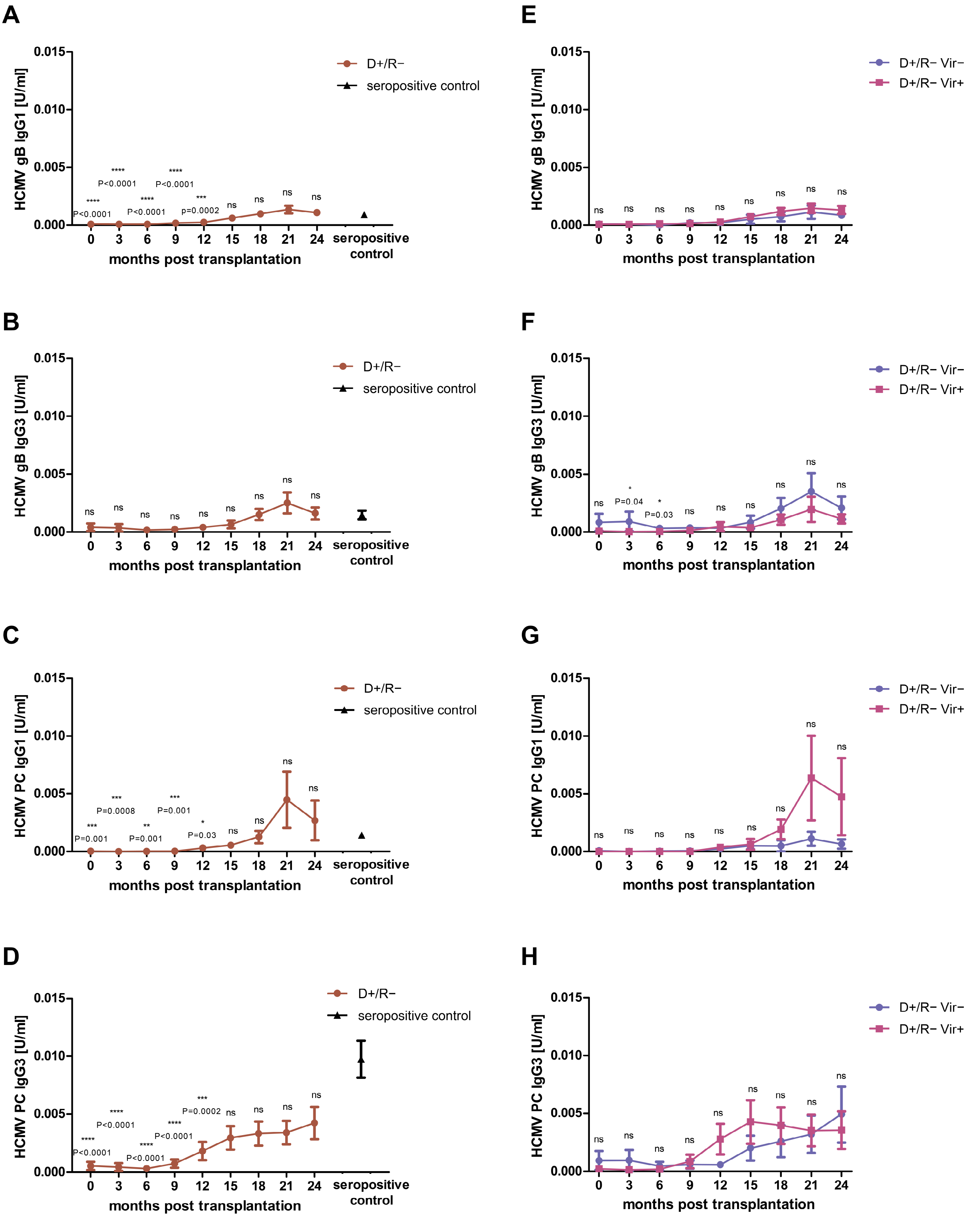

Figure S5. Analysis of HCMV-specific gB IgG1 titers (**A**,**E**), gB IgG3 titers (**B**,**F**), PC IgG1 titers (**C**,**G**), and PC IgG3 titers (**D**,**H**) in D+/R− lung transplant recipients (LTRs) after lung-transplantation (LTX). Patients were tested at 0, 3, 6, 9, 12, 15, 18, 21 and 24 months post LTX. Data are shown as mean antibody titers (± SEM). (**A**–**D**) Mean value (± SEM) of the HCMV-seropositive control cohort (N = 80) is indicated as black triangle. (**A**–**D**) Antibody levels at each time point of D+/R− LTRs were compared with those of the seropositive control patients ANOVA and Dunn's post-test. (**E**–**H**) Antibody levels at each time point of viremic (blue) (>1000 copies/mL) D+/R− LTRs were compared with those of non/low-viremic (pink) (<1000 copies/mL) D+/R− LTRs by Mann Whitney-Test; $p < 0.05$ was considered significant. *, $p < 0.05$; **, $p < 0.01$; ***, $p < 0.001$; ****, $p < 0.0001$. U/mL, units/milliliter; gB: glycoprotein B; PC: pentameric complex.

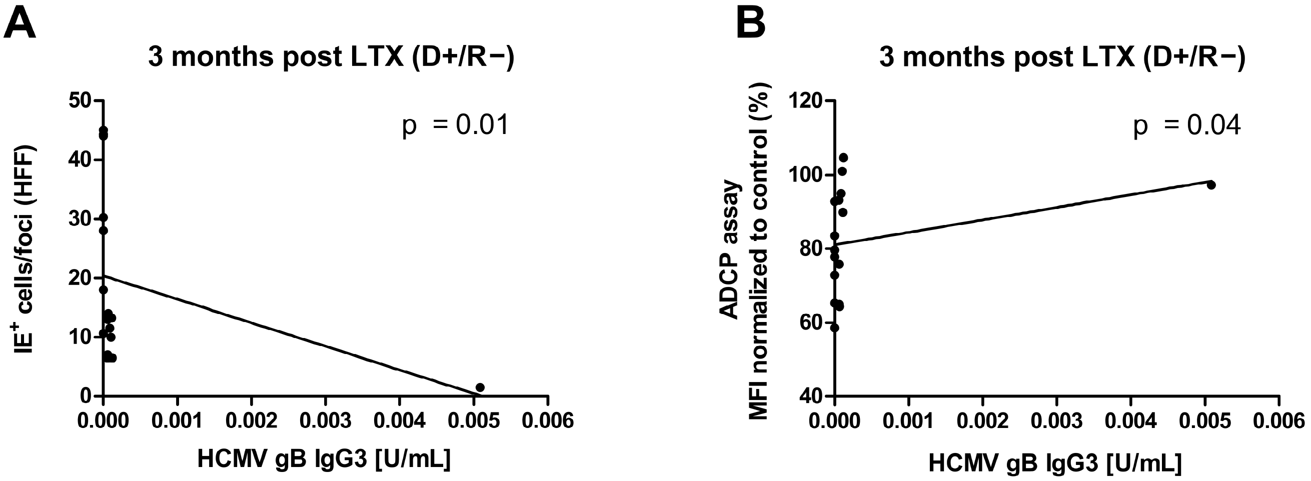

Figure S6. Correlation analysis of the HCMV-specific ADCP responses and HCMV-specific antibody titers in D+/R− LTRs. gB-specific IgG3 titers obtained at 3 months post-LTX were correlated with individual mean plaque sizes of Focus Expansion Assays (FEA) on fibroblasts (HFF) (**A**) or mean normalized mean fluorescence intensity (MFI) of antibody-dependent cellular phagocytosis (ADCP)-assays (**B**) at the same time point. Non-parametric two-tailed Spearman's rank correlation test was used to evaluate the correlation between HCMV-specific ADCP responses and gB-specific IgG3 titers. The solid black line indicates a linear regression. gB: glycoprotein B.

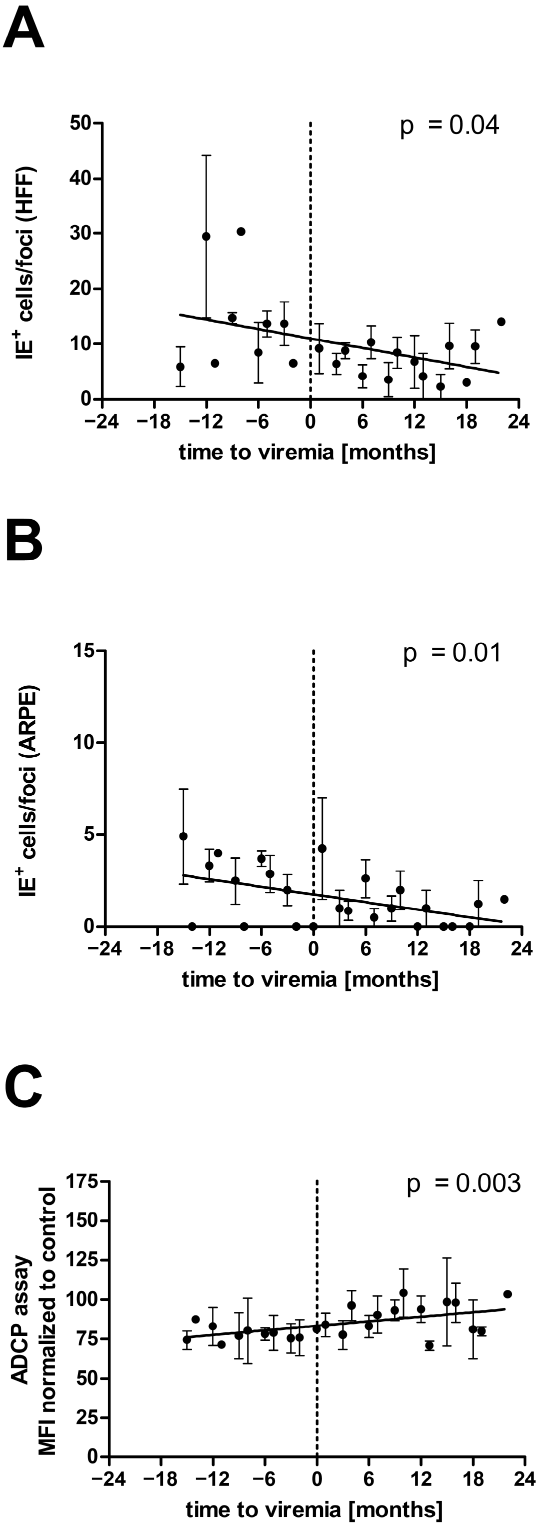

Figure S7. Analysis of the Focus Expansion Assay (FEA) on fibroblasts (HFF) (**A**), epithelial cells (ARPE) (**B**) as well the HCMV-specific ADCP response against cell-free virions (**C**) with plasma obtained from D+/R− highly viremic (>1000 copies HCMV DNA/mL) lung transplant recipients (LTRs) after lung transplantation (LTX). D+/R− LTRs were tested at 0, 3, 6, 9, 12, 15, 18, 21 and 24 months post LTX. Data are shown as mean IE^{+} /per foci (± SEM) (**A**,**B**) or as mean normalized mean fluorescence intensity (MFI) (± SEM) (**C**). Non-parametric two-tailed Spearman's rank correlation test was used to evaluate the correlation between HCMV-specific ADCP responses in the post-transplant follow-up. The solid black line indicates a linear regression. The dotted black line indicates the time of the first highly viremic episode.
